# Multimodality Video Acquisition System for the Assessment of Vital Distress in Children

**DOI:** 10.3390/s23115293

**Published:** 2023-06-02

**Authors:** Vincent Boivin, Mana Shahriari, Gaspar Faure, Simon Mellul, Edem Donatien Tiassou, Philippe Jouvet, Rita Noumeir

**Affiliations:** 1 CHU Sainte-Justine Research Centre, Montréal, QC H3T 1C5, Canada; vincent.boivin.3@ens.etsmtl.ca (V.B.); gaspar.faure@polymtl.ca (G.F.); simon.mellul@etu.univ-tours.fr (S.M.); edem.tiassou.hsj@ssss.gouv.qc.ca (E.D.T.); philippe.jouvet.med@ssss.gouv.qc.ca (P.J.); 2 Department of Electrical Engineering, Ecole de Technologie Supérieure (ETS), Montréal, QC H3C 1K3, Canada; 3 Department of Pediatrics, Université de Montréal (UdeM), Montréal, QC H3T 1C5, Canada

**Keywords:** clinical decision support system (CDSS), video database, depth sensor, children, infrared thermography, intensive care

## Abstract

In children, vital distress events, particularly respiratory, go unrecognized. To develop a standard model for automated assessment of vital distress in children, we aimed to construct a prospective high-quality video database for critically ill children in a pediatric intensive care unit (PICU) setting. The videos were acquired automatically through a secure web application with an application programming interface (API). The purpose of this article is to describe the data acquisition process from each PICU room to the research electronic database. Using an Azure Kinect DK and a Flir Lepton 3.5 LWIR attached to a Jetson Xavier NX board and the network architecture of our PICU, we have implemented an ongoing high-fidelity prospectively collected video database for research, monitoring, and diagnostic purposes. This infrastructure offers the opportunity to develop algorithms (including computational models) to quantify vital distress in order to evaluate vital distress events. More than 290 RGB, thermographic, and point cloud videos of each 30 s have been recorded in the database. Each recording is linked to the patient’s numerical phenotype, i.e., the electronic medical health record and high-resolution medical database of our research center. The ultimate goal is to develop and validate algorithms to detect vital distress in real time, both for inpatient care and outpatient management.

## 1. Introduction

### 1.1. Motivation

In children, 28% of vital distress events go unrecognized. These situations are associated with a tenfold increased risk of mortality [1,2]. Meanwhile, technological innovations and computing advances keep transforming health care. As such, assessment and real-time evaluation of distress events, i.e., respiratory, neurological, and hemodynamic distress, can benefit from such innovations [3,4,5,6,7].

Over the past two decades, access, organization, analysis, and the use of generated data in the process of medical care have been widely adopted by many hospitals across the globe [8,9,10,11,12,13]. Intensive care units (ICUs) present an especially compelling case not only because of high-quality data but also due to the variety of treatments and interventions. Several commercial and non-commercial ICU databases have been developed, typically recording data such as patient demographics, free-text notes and reports of different specialties, diagnoses or procedures, laboratory analysis results, vital signs, advanced monitoring data, ventilator parameters, operating parameters of complex equipment such as perfusion pumps, dialysis monitors, extracorporeal membrane oxygenation systems, and information in the form of images, audio, and video [8,11,14,15,16,17].

Intensive care units are work environments with increasing patient complexity and volume, emerging new technologies, and staffing shortages that are challenges to the provision of high-quality care [14,18,19]. In such environments, focus has shifted to improvement strategies and reorganization of care to optimize efficiency and quality of care, as well as providing an ideal environment to develop technologies applicable to outpatient management. Databases collected from ICUs have been perceived as a way of motivating clinical investigations offering exciting opportunities for research. They enable the development of clinical decision support tools as well as improving clinical research. They also promote capabilities, extract new knowledge, obtain guidance for improved patient care, and permit the testing of algorithms with real-world data [13,14,15,18,20].

Patients in critical conditions or those who require special care need to be closely monitored to intervene in cases of sudden worsening. Continuity of care is achieved through the monitoring of vital signs by bedside monitors and by intermittent human observation. Conventional vital sign monitoring technologies require the attachment of adhesive electrodes or transducers to the patient’s skin with wires. Monitoring with wired sensors is especially cumbersome for patients in neonatal intensive care units (NICUs) and pediatric intensive care units (PICUs). Furthermore, it is susceptible to motion artifacts, can cause discomfort and irritation, may damage the skin, increase the risk of developing an infection, and might interfere with clinical and parental care [21,22,23,24,25,26]. 

Likewise, children under two years of age are unable to express what they are feeling verbally, nonverbally, or deliberately through hand gestures, or head nods and head shakes. As a result, the rate and range of movements or facial expressions are considered to be indicators of potential abnormal motor patterns, pain, discomfort, and sedation levels in young children [5,23,25,27,28,29,30]. Clinical interventions, routine care events, and visual inspections are needed to evaluate abnormal movements or comfort levels in NICUs and PICUs and even by parents at home. However, accuracy is subjective and depends on the clinician’s expertise and healthcare resources; it is time-consuming and non-continuous and invokes high costs.

Over the past few years, video-based monitoring has been used to assist clinical staff in patient care and to access patients’ conditions. Video-based technologies are desirable due to their affordability and ease of mounting. These technologies are non-invasive monitoring solutions under investigation in NICUs and PICUs. However, to the best of our knowledge, there is no publicly available database for non-contact continuous physiological monitoring, and pain/discomfort assessment in the PICU.

To bridge this gap, the research center of the Centre Hospitalier Universitaire Sainte-Justine (CHU Sainte-Justine) de Montréal is creating a real-time monitoring and diagnostic system through videos for patients in the PICU and plans to include it in stand-alone devices for use in outpatient management. While most databases comprise only one modality of information, our video database is multimodal. This database incorporates thermographic, point cloud, and color videos to address hemodynamic changes, surface and volume calculations, and recognition and detection problems, respectively. Furthermore, this database is linked to our high-resolution research database and electronic medical records of patients [18,19,31,32,33].

The purpose of this paper is to describe the data acquisition system for images and videos from a PICU room to a secure research electronic database. The entire process, from the acquisition of the images and videos to the insertion of the data in the database in a structured way is done automatically and securely. The final framework has different components including (1) Data Collection and Synchronization, (2) Database Organization and Automatic Insertion of Data (3) User Interface Web Application and (4) an Authentication and Authorization component. The ultimate goal is to develop and validate algorithms to detect vital distress in real time, both for inpatient care and outpatient management.

### 1.2. Related Works

Vital signs that are usually monitored include heart rate (HR), respiratory rate (RR), blood pressure (BP), temperature (T), and peripheral oxygen saturation (SpO_2_). Non-contact monitoring is becoming the preferred solution in NICUs and PICUs, where patients need to be attached to multiple wired sensors. These sensors are uncomfortable, irritating, and prone to false alarms due to motion artifacts.

Villarroel et al. [24] recorded a total of 426.6 h of video from 30 preterm infants in the NICU in order to evaluate the accuracy and proportion of time that HR and RR can be estimated. In studies [3,26,34,35] Red Green Blue and Depth (RGB-D) sensors were used to estimate and assess the respiratory activity of neonatal and pediatric patients. Two RGB-D cameras (Kinect V2) were used in [3] to provide quantitative measures of tidal volume and RR in a PICU room. One Asus Xtion Pro Live Motion RGB-D was mounted over the cribs of three preterm infants in an NICU to estimate the RR by measuring morphological chest movements [34]. In [26,35] data were collected from an overhead Intel RealSense SR300 RGB-D camera in an NICU room for RR estimation of four and five patients, respectively. In a pilot study, different settings of the Asus Xtion Pro Live Motion RGB-D sensor were used to visualize and quantify the motion of the thorax and abdomen regions of a mannequin during the breathing process [4]. Chen et al. [36] proposed a non-contact HR monitoring system for neonates using an RGB camera, while Kim et al. [37] proposed the use of a thermal camera to estimate HR remotely for athletes and children. [6,7] assessed the efficacy of thermographic profiles to monitor changes in cardiac output and detect cardiogenic shock in post-cardiac surgery infants using RGB and thermal cameras (Kinect Azure and the FLIR Lepton 3.5 LWIR). Shi et al. [38] explored the use of an RGB-D sensor and an infrared thermal camera for the detection of neonatal necrotizing enterocolitis. They used the Kinect Xbox One sensor to capture RGB-D information and the FLIR A320 IR camera to record the thermal data of 12 neonates, each for 60 s.

Video-based monitoring is not limited to the vital signs and physiological assessment of patients. Throughout patients’ stays in critical and intensive care units, most of their time is spent in bed. Thus, monitoring body movement and facial expression leads to continuous and consistent monitoring of pain and abnormal movements in clinical environments.

An automatic movement analysis for preterm infants using an RGB-D sensor was proposed in [28]. The camera was placed over the infant lying on the crib to collect sequences of depth images. The detection of abnormal paroxysmal events in newborns, namely, clonic seizures and life-threatening apnea events using multiple video sensors placed around a patient was investigated in [23]. Their motion analysis approach was versatile and allowed them to investigate various scenarios, including a single RGB camera, an RGB-D sensor, or a network of a few RGB cameras. Refs. [39,40,41] focused on assessing pain and discomfort from facial expression and motion analysis. Sun et al. [40] used a fixed-position high-definition camera (uEye UI-222x) to evaluate 183 video segments of 11 infants to recognize infants’ status of comfort or discomfort. Refs. [39,41] utilized publicly available databases for assessing neonatal pain. 

### 1.3. Summary of Main Contributions

**Figure 1:**
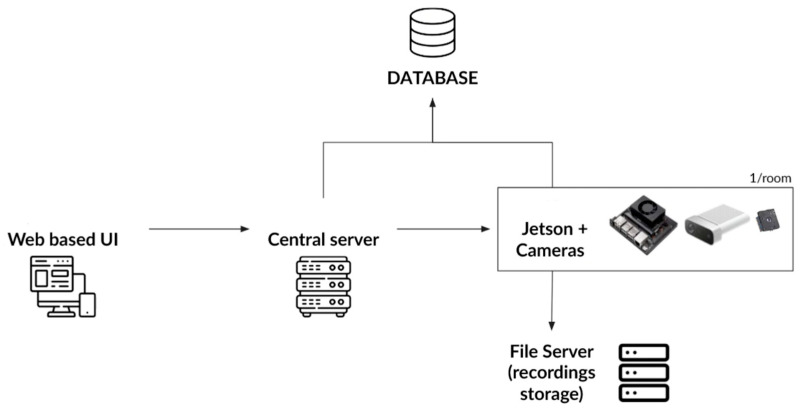
General architecture of our acquisition system with its components.

This work describes the architecture, design, and implementation of the first and second tiers of a data science pyramid, data collection and data storage, using the cyber-infrastructure setting of a public academic hospital. In short, this system captures videos and images from different cameras remotely and automatically, generates the associated metadata, and saves data in a structured way, all through a secure and easy-to-use web-based user interface (UI) accessible through the hospital intranet. Figure 1 depicts the general architecture of this distributed system with all its components.

To this end, three types of video data are collected: thermographic or infrared (IR) to provide heat distribution (mostly due to hemodynamic status), point cloud to enable surface and volume calculations (such as tidal volume), and Red Green Blue (RGB) or color data for general computer vision tasks such as detection and recognition. Cameras are calibrated prior to data collection, and these three types of video data are synchronized to ensure frame consistency before being stored automatically on hospital servers. 

The specific contributions can be summarized as the design and implementation of:

1. An acquisition software as well as an application programming interface (API) for the remote collection of video and image data from different cameras located in each PICU room;
2. A calibration and synchronization software to calibrate and synchronize data gathered through various devices;
3. A database that allows you to save data in a structured way using a database management system;
4. A software for automatic insertion of video and image data for patients admitted to the intensive care unit;
5. A web-based user interface (UI) and user experience (UX) to display recordings accessible both remotely and through the hospital intranet;
6. An authentication and authorization component that controls and communicates between multiple instances of the acquisition system in 1 and the web-based UI in 5;
7. A secure system accessible remotely with the above subsystems that can be deployed on different servers.

Objectives 1 through 6 are discussed in detail in Section 2: Material and Methods. Objective 1 is covered in Section 2.1 Data Collection, Objective 2 is reviewed in Section 2.2 Data Synchronization, Objectives 3 and 4 are discussed in Section 2.3 Database Organization and Interpretation, Objective 5 is examined in Section 2.4 User Interface Web Application, and Objective 6 is studied in Section 2.5 Authentication and Authorization. Objective 7, i.e., security and deployment of the final distributed system as well as the results, are discussed in Section 3: Results. 

## 2. Materials and Methods

### 2.1. Data Collection

Video and image data collected in this project are IR, point cloud, and RGB data. The IR data is gathered with a Flir Lepton 3.5 Long Wave InfraRed (LWIR), whereas point clouds and RGB are captured with an Azure Kinect development kit (DK).

Azure Kinect DK and its multiple sensors allow capturing raw depth (D) data as well as RGB videos. Acquisition of these two types of data is done through the Azure Kinect SDK (software development kit) interface and the Py4KA Python 3 library [42]. The Py4KA Library is used to start recordings as well as access raw data associated with recordings. Raw depth (D) data is post processed into point cloud data using Open3D [43] and Open Computer Vision (OpenCV). OpenCV adds color to the points, whereas Open3D is used to create the PLY file representing the point cloud on disk. 

To capture thermographic data, a Teledyne Flir Lepton 3.5 LWIR with a resolution of 160 × 120 pixels and a thermal sensitivity of ≤50 mK is used. To interact with the Flir Lepton device, the Flirpy library [44] is used. This allows the device to start recording and extract the data. There are two different types of data extracted using Flirpy: the raw thermal image and a matrix of the temperature for each pixel. The raw thermal image is stored directly on disk for later use, whereas the temperature matrix is saved in Kelvin in an Excel file using the Pandas library and the Openpyxl library [45].

For calibration of the two cameras, the RGB images from the Azure Kinect DK are superimposed with the IR images from the FLIR Lepton. This involves taking photos of a calibration marker whose dimensions are known [46]. Images of this marker are taken from both cameras, and the checkerboard is detected in each image using object detection. A special marker made of aluminum squares instead of ink is used for the IR sensor, so that the squares show up clearly in the IR image. As aluminum has a low IR emissivity, it shows up dark in the image, while the rest of the marker remains white.

**Figure 2:**
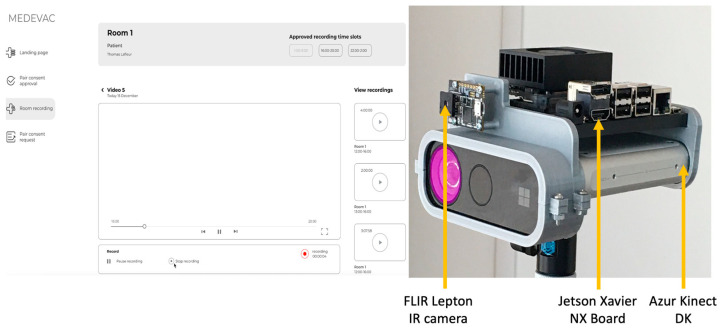
The multimodal video system. (**Left**): user interface used to record videos. (**Right**): hardware components including the Azur Kinect DK, FLIR Lepton IR sensor, and Jetson Xavier NX board.

These two cameras are connected to a Jetson Xavier NX board. The device is responsible for gathering raw data to process and produce all the artifacts, such as point clouds and Excel sheets with the temperature of each pixel for the thermographic data. In addition to data processing and recording, the acquisition software provides an API to command the Azure Kinect DK and FLIR Lepton to start recording remotely. The API is created using the REST API to connect the software on the Jetson Xavier NX to other devices running on the same network. Since most of the operations executed by acquisition software are I/O related, the asynchronous server gateway interface (ASGI) web server framework [47] is used for the REST API. To reduce the load on CPU while recording, data is processed after each recording. This is implemented with the in-process background task of FastAPI, which enqueues the captured data with the desired processing method to be completed later. The hardware setup and data acquisition UI used for this project are shown in Figure 2.

### 2.2. Data Synchronization

Raw data is gathered through different sensor interfaces. This results in these three types of data being unsynchronized. Unsynchronized data can be used independently, yet it is not suitable for other applications such as machine learning. Consequently, it is non-trivial to synchronize data between the Azure Kinect DK device and the Teledyne Flir Lepton device. The FLIR Lepton can record at a framerate of up to 9 frames per second (FPS), while the Azure Kinect can record up to 30 FPS. In other words, for every frame captured by the FLIR Lepton, there would be roughly 3 (30/9) frames captured by the Azure Kinect DK.

To synchronize data between the Azure Kinect DK device and the Teledyne Flir Lepton 3.5 LWIR device, the timestamps of their frames can be compared. However, due to the granularity of timestamps and the FPS difference between the two devices, comparing timestamps of frames is not an optimal approach for synchronization. In Table 1, the number of synchronized frames using the timestamps approach is reported. The best reported *Ratio of Frames Saved over Total Frames Captured* is only 0.68% (4 out of 585 frames) for a duration of 15 s. Since timestamps are in nanoseconds and are relatively small, this approach is unsuitable for synchronization.

To improve the ratio of synchronized frames, the “windowing query” approach, also known as the “interval problem approach”, is used. This approach is popular in scheduling and meeting applications for which the availability of someone in a time interval for a specific day needs to be determined. For this project, a mutable, self-balancing interval tree is used [48]. This approach stores time intervals and is optimized for the intervals overlapping with a given interval query. To collect all overlapping data from Azure Kinect DK and FLIR Lepton, the time window at the higher frame rate is selected, i.e., $1/maximum\;\left(FPS\right)$. This is the optimal time window for this project: in addition to synchronizing all overlapping frames within the same time interval, additional frames from the faster-producing device are collected.

Table 2 presents synchronization results using the “intervaltree” approach and the time window of $1/maximum\;\left(FPS\right)$. Here, the reported *Ratio of Frames Saved over Total Frames Captured* is 62% at the lowest and 72% at the highest. It is important to note that this number is higher than 33% (30/9) because the device with the higher frame rate might have a frame without corresponding frame from the device with the lower frame rate. In this case, we still save those frames in the database to keep the data from the faster-producing device.

### 2.3. Database Organization and Interpretation

To create a database that allows saving raw and post-processed synchronized data in a structured way, PostgreSQL is chosen. It is an open-source database management system (DBMS) capable of managing large amounts of data as well as supporting several operating systems. The data to be stored includes not only videos and images but also all the associated metadata. This includes an exceptionally large amount of data that must be saved in a structured way so that the metadata can be associated with the right image or video.

To create the database, initial, data attributes are specified. Our research team decided that patient ID, patient’s consent, video URL, metadata, video acquisition parameters, video calibration parameters, and annotation to the video are the most important attributes. These data attributes are critical as the database is meant to be used for research and diagnostic purposes in later stages.

**Figure 3:**
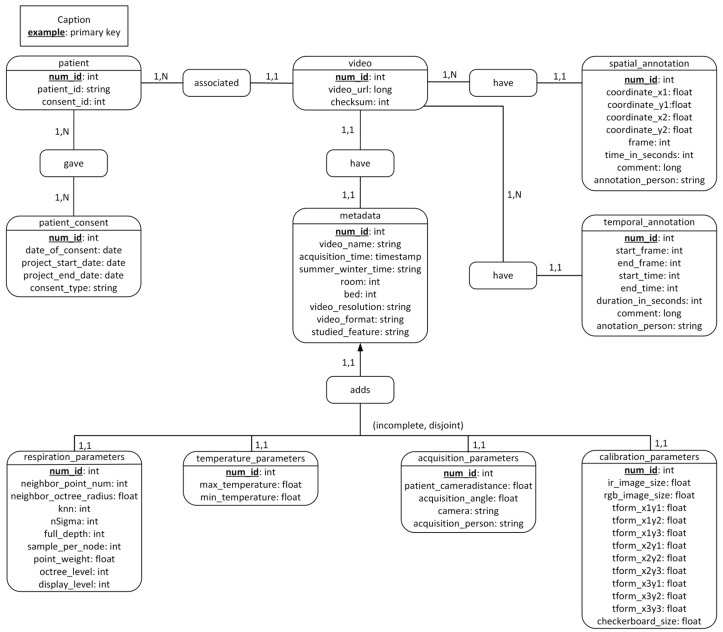
Database Conceptual Model: the overall view of concepts or entities with respect to their relationships in our database.

Based on the above attributes, the conceptual data model is built. Figure 3 showcases different tables in the database and illustrates the relationships between cardinalities. The final tables are *patient, patient_consent*, *video*, *metadata*, *respiration_parameters*, *temperature_parameters*, *acquisition_parameters, calibration_parameters*, *temporal_annotation*, and *spatial_annotation*.

The *patient* table is the one that stores the patient identifier. This identifier is not the unique identifier of a patient that is used in their electronic medical record (EMR), but a temporary number used only in the context of video acquisitions. The purpose of this identifier is to maintain the confidentiality of patients. A secure system is in place to link this identifier to the patients’ unique identifier found in their health records. This table also contains the identifier of the patient’s consent, which makes it possible to specify which type the patient or their parents have consented to: data storage for research purposes only or consenting to the use of videos for scientific presentations.

We created two more tables, *temporal_annotation* and *spatial_annotation*, for potential annotations to the video that can be inserted later. *temporal_annotation* is used to comment on a temporal range of the video, whereas *spatial_annotation* is used to specify the coordinates of the region of interest for the image. In the *temporal_annotation* table, we can find attributes that allow us to define the beginning and end of the video sequence to be commented. In the *spatial_annotation* table, we can find the coordinates that allow us to annotate a zone of the image as well as which image in the video we are annotating. For both types of annotation, the person who made the annotation is specified.

The *metadata* table gathers all the data related to the videos, such as the date and time of acquisition, the name of the video, and the resolution. *respiration_parameters* and *temperature_parameters* are two separate tables that complement the metadata table. Temperature and respiration information would record some specific data whenever necessary; thus, the information in these two tables is not necessarily logged when recording a video. Nevertheless, their information is related, so two new tables specific to the type of acquisition are created.

In addition, the cameras that make these videos have calibration and acquisition parameters that are worth saving. Thus, the *calibration_parameters* and *acquisition_parameters* tables are created. Saving all this data will allow researchers to compare different acquisitions and draw conclusions.

**Figure 4:**
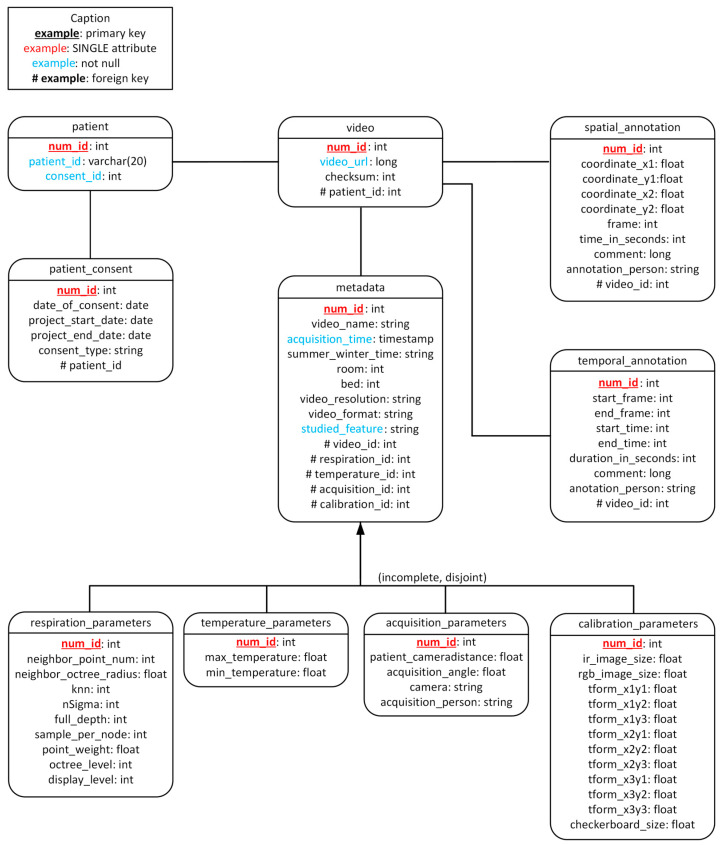
Database Relational Model.

Finally, the conceptual model (Figure 3) lets us build the relational model (Figure 4). The relational model defines the relationships between tables more precisely. These two models are the basis for creating the database with PostgerSQL.

For the automatic insertion of data, Java is used. PostgreSQL is an object-relational database management system (ORDBMS), which means that data is managed and represented as objects. As such, when there is a new occurrence of any of the events, it is inserted and validated into the directory/database automatically. This means that the application manages to recognize when a new file is created, retrieve the file path, and send it to the class that reads the data. In the same way, the results are used to validate that the writing of the SQL query and the sending of it by the Java Database Connectivity (JDBC) driver are done correctly.

Sending SQL queries in the usual unprotected way has flaws and some vulnerabilities because, when the Java application communicates with the DBMS without protection, additional query snippets can be added to the base query without being expected. Using the NamedParameterJdbcTemplate library [49] allows one to work with queries that are parameterized. This allows for data validation and solves this security problem. This is especially important in this project because we are working with patient data that requires a high degree of confidentiality.

### 2.4. User Interface Web Application

User Interface (UI) is responsible for showing a preview of each room and displaying recordings through a video stream. However, this web-based UI would not interact directly with Data Collection and Synchronization’s API; rather, it would interact with the authentication and authorization system. The reason for this will be discussed later in more detail in Section 3 and Section 4: Results and Discussions.

The web-based UI design should account for the user’s minimal or nonexistent interactions with the data acquisition system. We chose React [50] for the web-based UI; it is a single-page application (SPA) UI library and is responsible for doing the task of displaying the content to users. React is open source, well-documented, has plenty of styling libraries to choose from, and separates concerns between the server and the client. The issue with React is that SPAs are created with a framework that involves a lot of JavaScript. This means that the user’s browser needs to download the JavaScript code of the chosen library before the website can interact with or display it to the user. We use a React framework called NextJS [51], as NextJS abstracts all the complexity of server-side rendering with React while allowing the user to see the website while the required components and libraries might still be loading. To make the web-based UI visually appealing, a React UI library called Material UI (MUI) is used [52].

The other important consideration in developing a web-based UI is enabling it to display different data types to the user. Displaying IR data or RGB data is like displaying an image. To reduce the network bandwidth required to display RGB videos, the video stream is compressed before being previewed. The JPEG method in OpenCV is used to compress the RGB images.

However, displaying point cloud data differed in two ways: the file size and the type of data itself. This 3D data is saved as a PLY file on the Jetson Xavier. These PLY files represent points in 3D space with extra metadata, such as the color of each pixel, when available. With a resolution of 256 × 256 pixels, each file weighs around 38 MB. This size could impact the hospital’s internal network, so each PLY file needs to be compressed before being displayed. To this end, a JavaScript Object Notation (JSON) file is created, which contains two arrays: points and colors. The points array contains the coordinates of all the points in 3D space, and the colors array has the color data for that point. Since JSON files are text files, they can be compressed with any traditional compression algorithm. The compressed JSON file size (around 3 MB) can be safely transferred over the internal network and displayed using ThreeJS [53] and React Three Fiber [54].

**Figure 5:**
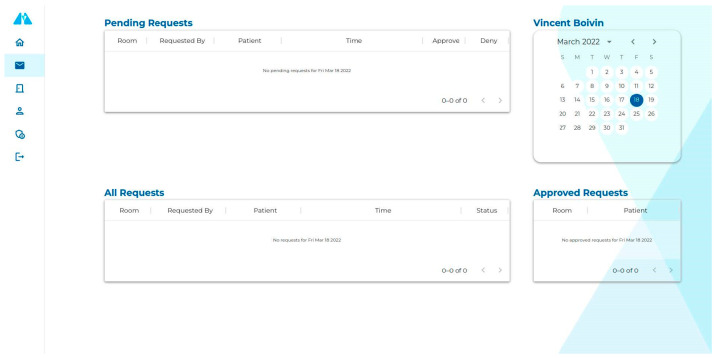
A screenshot of the web application UI on a desktop computer.

**Figure 6:**
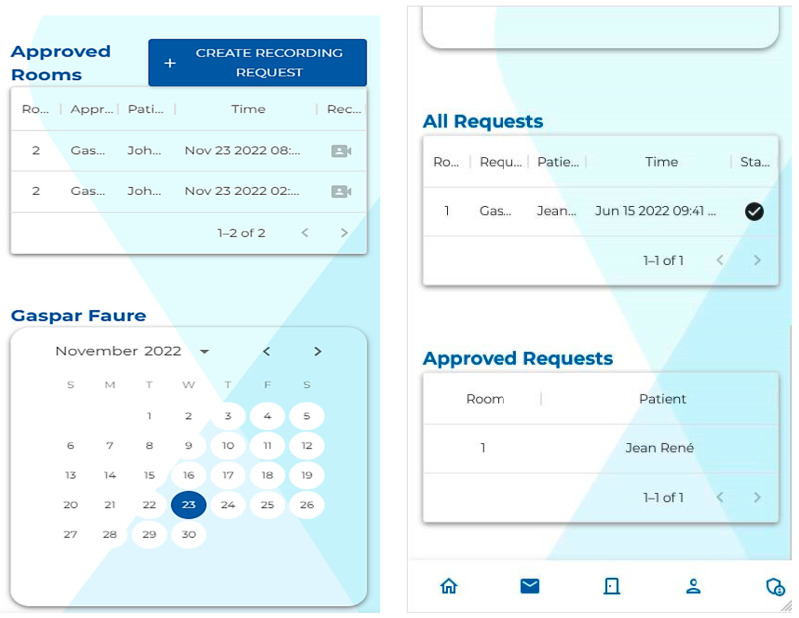
A screenshot of the web application UI on a mobile device.

Creating a web application to interact with the API allows for the development of a modern user interface. As we can see in Figure 5 and Figure 6, the user interface remains similar while adapting to the screen size. This makes it easier for the user to transition from one device to another. This also allows the user to render 3D point clouds directly in the browser, i.e., the user does not have to use any specialized 3D software to see the recorded data.

### 2.5. Authentication and Authorization

The authentication and authorization system acts as the communicator as well as a secure connection between the web-based UI and multiple instances of the data acquisition system.

Having an intermediary system or an API gateway is necessary as data acquisition might be running in more than one room and on more than one of the Jetson Xavier NX devices. The PICU at CHU Sainte-Justine has 32 beds, and this system is installed in all these rooms. This intermediary system prevents web-based APIs from having to know and connect with every single instance of data acquisition directly. Additionally, an API gateway allows us to put a facade on all the other systems and make them seem like one coherent system. While this project does not have a microservice architecture, it could be seen as one with each embedded device as a microservice. In that context, each Jetson Xavier NX device would act as a microservice.

Nginx, an open-source software that can serve as a reverse proxy and a web server, is used to make the API gateway. This system is also responsible for non-camera-related requests such as authentication and authorization. To facilitate maintenance, the modules are separated: modules running on devices and connected to the cameras reside in a separate codebase that is distinct from the rest of the code, which is unrelated to the cameras.

The two APIs, are authenticated using OAuth2 [55] with JSON Web Tokens (JWT). The authentication API has two parts: the access token and the refresh token. The access token is a JWT, which contains data allowing the user to be identified. The refresh token is an encrypted string that allows the user to request another JWT token without signing in again. To prevent someone from stealing another user’s identity or refresh token, three security measures are put in place. The original IP of each user is kept in the database after each sign-in attempt. This IP is verified the next time the user asks for a new JWT with a refresh token. If IPs are not matched, the refresh token will be deleted from the database, and the user will be asked to sign in again. Furthermore, each JWT and refresh token would expire after a certain amount of time. In order to ensure both a secure system and a pleasant user experience, we have implemented fast-expiring JWTs alongside long expiring refresh tokens. 

The authorization API assigns and verifies user permissions. Any user can have multiple roles, such as Recorder, Approver, Inputter, and Admin. This API enables user verification as well as their permission. As such, if users are authenticated but do not have the required permission, their access is denied, and the server would deny the request with an HTTP status code of 401.

### 2.6. Ethics

The study and database construction were approved by the institutional ethics committee of Sainte-Justine Hospital (protocol code 2016-1242, approved on 31 March 2016). Parental consent is obtained by a research assistant with human ethics training prior to video recording. The exploitation of the database is regulated by a database policy validated by the institutional review board. Additionally, the physicians that labeled the data are medical students or attending physicians trained in the management of critically and acutely ill children.

## 3. Results

**Figure 7:**
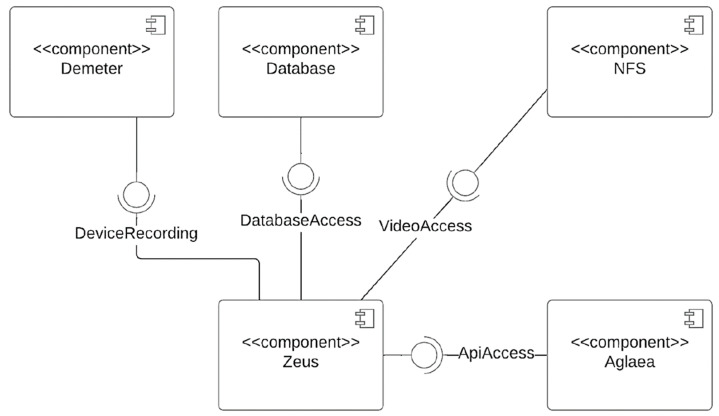
Component diagram of subsystems of the proposed distributed system.

The final framework of this project is depicted in Figure 7. The proposed system is a distributed system that focuses on efficiency and usability. As seen in Figure 7, it consisted of various subsystems, including a (1) Data Collection and Synchronization component, (2) Database Organization and Automatic Insertion of Data (3) User Interface Web Application and (4) an Authentication and Authorization component. The software for data collection and synchronization, as well as 3D rendering in the browser, was developed specifically for this project. The other two are off-the-shelf: the database component uses PostgreSQL, and the network file system (NFS) component uses Windows Server. The web-based API never interacts directly with the software running on the cameras. Instead, there is an API gateway between those two components. This design choice allows data acquisition and synchronization to solely focus on features regarding the camera, whereas the authentication and authorization system could focus on security and HTTP communication.

**Figure 8:**
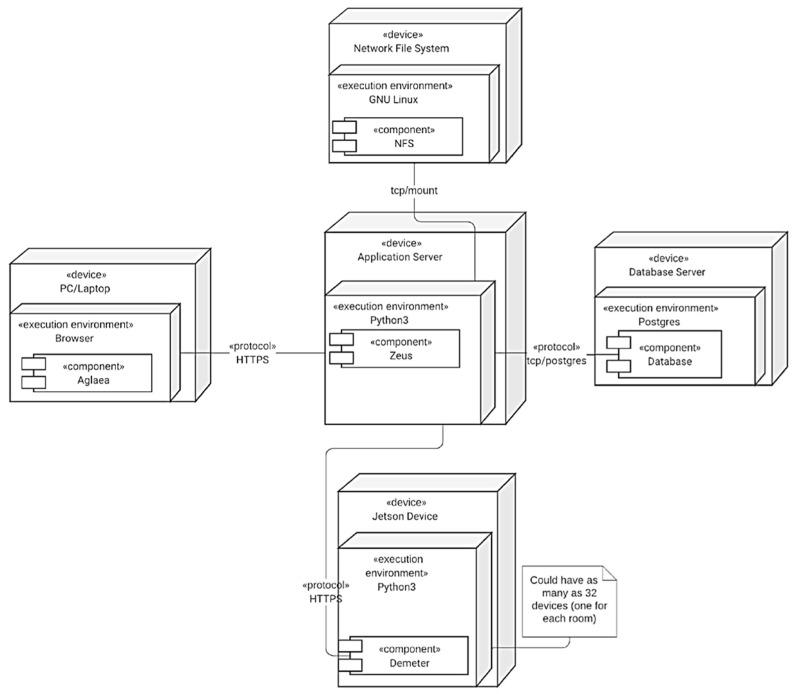
Deployment diagram of subsystems of the proposed distributed system on different servers.

These components are not all deployed on the same server (Figure 8). There is only one instance of the authentication and authorization system deployed on the server. As for data acquisition and synchronization, Azure Kinect DK, Flir Lepton 3.5 LWIR, and Jetson Xavier NX boards are used. The data acquisition and synchronization component has 32 instances, one deployed for each PICU room. Finally, the server-side rendering component is on the same server as the authentication and authorization system. As for the client-side component, it does not have to be deployed at all; it will be downloaded over the wire by each user when they first load the web application. 

The results of the synchronization technique save up to 72% of captured frames (ratio of frames saved over total frames captured coming from two different cameras) while still having the frame synced within the time window defined (see Table 2). The recording also included all the necessary metadata to enable the correct usage of the point cloud, IR, and RGB data.

Moreover, the data collection and synchronization component and the authentication and authorization component are easily accessible through REST APIs and WebSocket. This setup allows the development of UI web-based applications and facilitates the use of acquisition components related to the cameras. With this, a user could perform recordings without having a deep knowledge of the systems used by the cameras.

Containerization is used for the web-based UI as well as the authentication and authorization system’s API to facilitate transfer and deployment in different environments. Data collection and synchronization’s API is not containerized because it needs extra configuration to access devices connected through USB ports. As such, each of these components runs on different web servers. The web-based API is easy to containerize, as NextJS provides a quick way to deploy the application with containers. It does not require any access to disk; it is a front-end application and does not need access to any file on the system on which it is running. The authentication and authorization system bootstraps several components, such as the database running PostgreSQL, the reverse proxy, and itself. PostgreSQL needs access to the disk to have persistent data. As for the reverse proxy, volumes are used to access files such as the configuration files and the public and private keys used for HTTPS communication. Finally, the system itself needs access to the disk to be able to save log files.

This deployed system can be commanded securely and remotely through an API to start recordings in any of the rooms. Raw data is post processed into point clouds and RGB, as well as the raw thermal image and a matrix of the temperature of each pixel. It is synchronized according to a time window before being stored automatically on the research servers.

The recordings in this database started on 14 April 2021, and to date, there are more than 290 recordings in the database. Each recording is a 30 s long video for any of the RGB, IR, or point cloud videos. Data collected in the research database includes the patient’s specific identification number as well as the data described above. From admission to discharge, all patients’ demographic, physiologic, medical, and therapeutic data are prospectively collected. All this information is linked to our high-resolution research database and electronic medical records for patients. 

To further enhance data security, the servers dedicated to the database are physically located in the informatics department of the CHU Sainte-Justine with restricted access. The applied clinical research unit of the hospital oversees the database and workstation maintenance and security.

## 4. Discussion

This article described a video acquisition system in a 32-bed medical, surgical, and cardiac PICU at a free-standing tertiary maternal and child health center, i.e., the Centre Hospitalier Universitaire Sainte-Justine (CHU Sainte-Justine) de Montréal, QC, Canada.

The use of video and image databases have been increasing because of the value they add to the assessment of vital distress. Our proposed system has the potential for effective patient monitoring as well as facilitating clinical decision-making at the bedside. Having such a video database is crucial for the development and validation of algorithms to quantify and standardize vital distress in children. Since this database is linked to a patient’s numerical phenotype [18,19,31,32,33], we can use video data with demographic and biological data to develop clinical decision support systems. This work was the first step to making this possible. Accordingly, the main goal of this project was to automate the whole process in a secure way, from the acquisition of the images and videos to the insertion of the data in a database in a structured way. 

Video data collected in this work were thermographic or infrared data (IR), point clouds, and Red Green Blue or color (RGB) data. IR data provided heat distribution, point clouds enabled surface and volume calculations, and RGB addressed different computer vision tasks. The IR data was collected with a Teledyne Flir Lepton 3.5, whereas raw RGB-D (RGB and depth) data was captured with an Azure Kinect DK (development kit). Raw depth data was post-processed into point cloud data using Open3D [43] and OpenCV. Both sensors (Teledyne Flir Lepton 3.5 and Azure Kinect DK) were connected to a Jetson Xavier NX board and were calibrated prior to data collection. The three types of video data, i.e., IR, RGB, and point cloud, were synchronized to ensure frame consistency before being stored automatically on hospital servers. Moreover, a web application as well as an authentication and authorization system were developed to make recording secure and remotely accessible. Cameras were installed on the ceiling, above each PICU bed. As such, the design for sensor configuration and the data acquisition process did not have an inference with regular patient care.

A system was created as a result, which had the following subsystems: (1) Data Collection and Synchronization component, (2) a Database Organization and Automatic Insertion of Data component (3) User Interface Web Application component and (4) an Authentication and Authorization component. The choices of frameworks and technologies such as Java, PostgreSQL, FastAPI, and Nginx were made based on efficiency and usability. PostgreSQL and Java were both openly available. FastAPI allowed us to build easy-to-use and well-documented REST APIs through its in-process background tasks, WebSocket support, OpenAPI documentation support, and ASGI by default feature. As for reverse proxy, the API gateway architectural pattern was implemented without any coding. Finally, the system had a distributed design, for which any component could be deployed on a different computer/server.

Any recording took place only if patients and/or their parents signed the consent forms. Cameras were commanded to perform a 30 s acquisition and to send recorded data automatically to the research database then. There were two types of consents for this project: those who agreed to have their data used for research purposes only and those who agreed on their data to be used for research as well as their data appearing in conferences and publications. The reason for admission is not considered, and any patient in the PICU who agreed to participate in this study was considered. We included all patients under 18 years old at admission to the PICU of CHU Sainte-Justine who consented to be part of this study.

On a final note, it is important to state that our database does have certain limitations. Firstly, data are collected from patients who agreed to be part of this study. As a result of this, the variability in our database may be limited to specific age groups and/or ethnic backgrounds. For this study, we included all patients from the age of 0 to 18 years at admission to the pediatric ICU of Sainte-Justine University Hospital. To date, the median age in our database is 6 months old. This could lead to bias in future research and studies. As data acquisition continues, we might have less bias toward a specific age group. Secondly, cameras are installed and fixed to the ceiling above each PICU bed. This configuration is to ensure that the data acquisition process does not interfere with regular patient care. Nevertheless, this setup provides only one viewpoint and limits the visualization of some signs of respiratory distress, such as thoraco-abdominal asynchrony. Furthermore, this database, like many others, is single centered. This limits its generalizability. For the generalizability of algorithms, there is a need to develop similar databases in other PICUs. With knowledge translation, this architecture can be replicated in other PICUs with similar networks. This allows for the potential for further data expansion.

## 5. Conclusions

The accomplished work was the creation of a clinical video database to support a wide range of clinical studies aimed at analyzing vital distress in children. This database has already been used in a few research works [3,4,5,6,7]. Reliable localization and tracking of the eye region for hospitalized PICU patients using RGB images has been achieved [5]. The future direction of this work is to assess pain and evaluate comfort in young children. Non-invasive heat distribution at the skin surface using infrared thermography (IRT) was studied in [6,7]. In the future, we can combine IR and RGB to evaluate the hemodynamic status of patients (such as clinical hypoperfusion). Analyses of respiratory distress as well as tidal volume have been studied in [3,4]. A future direction of these works is to investigate respiratory volumes (according to ventilation mode) by fusing RGB and point cloud data. Another future research direction is to use RGB and IR videos to analyze sleep posture and evaluate distressed motion (such as apnea and seizure) in children. Nevertheless, there are far more clinical and research applications for this database. 

The main question that needs to be currently addressed is the following: “Does this database have the potential to assist in clinical practice through the development of algorithms that monitor vital distress?”. To answer this question, the research challenges are: to record a wide range of videos for various clinical scenarios in pediatric patients (aged 0 to 18 years old); to check the reliability of the data collected; to develop real-time algorithms that analyze vital distress; to validate the possible use of algorithms in inpatient and outpatient care; to create similar databases in other PICUs to further generalize algorithms; and to create a multimodal multiview camera framework to fuse and incorporate observations from multiple modalities and views. Accordingly, the following steps are to check the reliability of the collected data and validate its possible use in inpatient and outpatient care. 

## Figures and Tables

**Table 1:** Number of synchronized frames saved using timestamps (in nanoseconds).

Duration of Recordings (Seconds)	Azure Kinect DK Framerate (Faster Device)	FLIR Lepton Framerate	Number of Saved Frames	Ratio of Frames Saved over Total Frames Captured
5	30 FPS	9 FPS	2	1.03% (2/195)
10	30 FPS	9 FPS	0	0.00% (0/390)
15	30 FPS	9 FPS	4	0.68% (4/585)

**Table 2:** Number of synchronized frames saved implemented with the “intervaltree” approach.

Duration of Recordings (Seconds)	Azure Kinect DK Framerate (Faster Device)	FLIR Lepton Framerate	Number of Saved Frames	Ratio of Frames Saved over Total Frames Captured
5	30 FPS	9 FPS	140	72% (140/195)
10	30 FPS	9 FPS	240	62% (240/390)
15	30 FPS	9 FPS	400	65% (400/585)

## Data Availability

The MEDEVAC database generated during the current study is not publicly available due to institutional restrictions on data sharing and privacy concerns. However, it is accessible for research purposes given the approval from the Research Ethics Board of CHU Sainte-Justine is obtained.
